# Two clinical drugs deubiquitinase inhibitor auranofin and aldehyde dehydrogenase inhibitor disulfiram trigger synergistic anti-tumor effects *in vitro* and *in vivo*

**DOI:** 10.18632/oncotarget.6425

**Published:** 2015-11-28

**Authors:** Hongbiao Huang, Yuning Liao, Ningning Liu, Xianliang Hua, Jianyu Cai, Changshan Yang, Huidan Long, Chong Zhao, Xin Chen, Xiaoying Lan, Dan Zang, Jinjie Wu, Xiaofen Li, Xianping Shi, Xuejun Wang, Jinbao Liu

**Affiliations:** ^1^ State Key Laboratory of Respiratory Disease, Protein Modification and Degradation Laboratory, Department of Pathophysiology, Guangzhou Medical University, Guangdong 511436, People's Republic of China; ^2^ Guangzhou Research Institute of Cardiovascular Disease, The Second Affiliated Hospital, Guangzhou Medical University, Guangdong 510260, People's Republic of China; ^3^ Division of Basic Biomedical Sciences, Sanford School of Medicine of the University of South Dakota, Vermillion, South Dakota 57069, USA

**Keywords:** auranofin, deubiquitinase inhibitor, disulfiram, anticancer strategy

## Abstract

Inhibition of proteasome-associated deubiquitinases (DUBs) is emerging as a novel strategy for cancer therapy. It was recently reported that auranofin (Aur), a gold (I)-containing compound used clinically to treat rheumatoid arthritis, is a proteasome-associated DUB inhibitor. Disulfiram (DSF), an inhibitor of aldehyde dehydrogenase, is currently in clinical use for treating alcoholism. Recent studies have indicated that DSF can also act as an antitumor agent. We investigated the effect of combining DSF and Aur on apoptosis induction and tumor growth in hepatoma cancer cells. Here we report that (i) the combined treatment of Aur and DSF results in synergistic cytotoxicity to hepatoma cells *in vitro* and *in vivo*; (ii) Aur and DSF in combination induces caspase activation, endoplasmic reticulum (ER) stress, and reactive oxygen species (ROS) production; (iii) pan-caspase inhibitor z-VAD-FMK could efficiently block apoptosis but not proteasome inhibition induced by Aur and DSF combined treatment, and ROS is not required for Aur+DSF to induce apoptosis. Collectively, we demonstrate a model of synergism between DSF and proteasome-associated DUB inhibitor Aur in the induction of apoptosis in hepatoma cancer cells, identifying a potential novel anticancer strategy for clinical use in the future.

## INTRODUCTION

Cancer cells have been shown to depend on the ubiquitin proteasome system (UPS) more than normal cells. The successful application of 20S proteasome peptidases inhibitor, bortezomib, approved by the United States Food and Drug Administration (FDA) for multiple myeloma treatment, established a therapeutic target based on UPS [1]. Unfortunately, the same as other chemotherapeutic agents, drug resistance has become an increasing concern and limits the administration of bortezomib [2, 3]. Therefore, alternative therapeutic strategies are required for cancer treatment.

Recently, a strategy based on inhibition of proteasome-associated deubiquitinases (DUBs) has emerged as a promising anti-cancer therapy [4–6]. In eukaryotes, DUBs remove the ubiquitin (Ub) and ubiquitin-like (Ubl) chain from target proteins prior to their degradation and thereby are involved in regulating multiple cellular processes, including cell cycle control [7, 8], DNA damage response and repair [9–11], chromatin modification [12], and various signal transduction pathways [13]. The human genome is found to encode approximately 100 putative DUBs, which are subdivided into six families according to their catalytic and structural features. Among these DUBs, POH1, UCHL5 and USP14 are associated with the 19S proteasome; they are often overexpressed in several carcinoma cells, which renders them potentially new therapeutic targets in these cancer cells [14–17].

Auranofin (Aur), a gold (I)-containing agent, is clinically used to treat rheumatic arthritis for more than 30 years. Recent studies have demonstrated that Aur has potent antitumor effects beyond its anti-inflammatory activity [18, 19]; therefore, it has been approved by FDA for Phase II clinical trial in cancer therapy (http://clinicaltrials.gov/ct2/show/ NCT01419691). Several potential mechanisms were proposed for the anti-cancer effects of Aur, including inhibition of thioredoxin reductase (TrxR), over generation of reactive oxygen species (ROS), loss of mitochondrial membrane potential (MMP), and induction of endoplasmic reticulum (ER) stress and caspase activation [20–22]. However, we have recently unraveled that Aur inhibits 19S proteasome-associated DUBs (mainly UCHL5 and USP14), accumulates ubiquitinated proteins (Ub-prs), and induces unfolded protein response (UPR) followed by cell apoptosis. Additionally, our previous studies have suggested that Aur can stimulate cellular ROS generation but this is not required for Aur to induce apoptosis [23–25].

Disulfiram (DSF) is currently in clinical use for the treatment of alcoholism by irreversibly inhibiting aldehyde dehydrogenase. Several studies have shown that DSF possesses an anticancer activity in various cancer cells [26–28]. In addition, it was reported that DSF, as a cooper-binding agent, induced apoptosis in breast cancer via proteasome inhibition [29]. It was also reported that DSF, when complexed with copper, could induce ROS-dependent apoptosis of prostate cancer cells [30]. Moreover, it was reported that DSF and its metabolites could be used as a chemosensitizer of some anti-cancer agents [31].

Here we report that the combination of Aur and DSF synergistically enhances their cytotoxicity and cell apoptosis of hepatoma cancer cells in both cultures and xenograft models and the synergistic effect is associated with enhancement of proteasome inhibition, induction of ER stress, loss of MMP, and caspase activation.

## RESULTS

### Aur and DSF synergistically inhibit cell proliferation and colony formation of both SMMC-7721 and HepG2 cells

**Figure 1:**
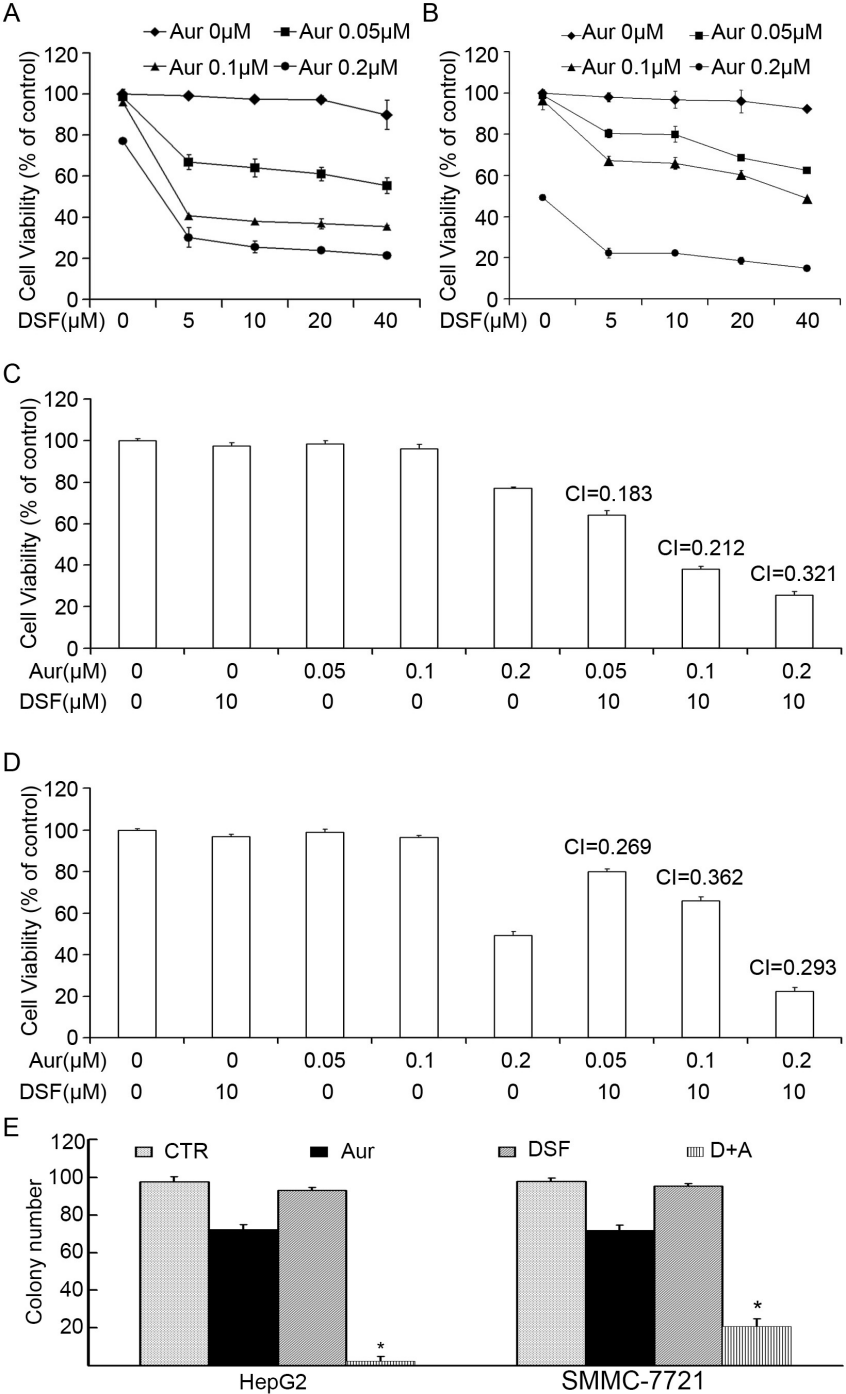
Combination of Aur and DSF synergistically reduced cell viability *in vitro* (**A**) and (**B**) HepG2 or SMMC-7721 cells were treated with the indicated concentrations of Aur, DSF or the combination for 48 h. Cell viability was detected by MTS assay. Mean ± SD (*n* = 3). DSF, Disulfiram; Aur, Auranofin. (**C**) and (**D**) Combination index (CI) was shown. CI < 1 indicates synergism; CI = 1 indicates additive effect; and CI > 1 indicates antagonism. HepG2 or SMMC-7721 cells were treated with either DSF (10 μM), Aur (0.05, 0.1, 0.2 μM) or the indicated combination for 48 h. (**E**) HepG2 and SMMC-7721 cells exposed to Aur (0.2 μM), DSF (10 μM) or their combination for 12 h were suspended in 30% agarose for 7 days, colony formation was counted. *P < 0.05, compared with other treatments.

To determine whether DSF sensitizes cancer cells to Aur treatment, we first tested the effect of various concentrations of DSF (5, 10, 20, 40 μM) or Aur (0.05, 0.1, 0.2 μM) alone or in combination on the cell viability of human hepatocarcinoma HepG2 and SMMC-7721 cells using the MTS assay. We found that treatment with either DSF or Aur alone for 48 h only slightly reduced the cell viability but a dramatic decrease of viability was induced by the co-treatment at each concentration (Figure 1A and 1B). The combination indices of the treatment of 10 μM DSF combined with 0.05, 0.1, or 0.2 μM Aur in hepatocarcinoma HepG2 and SMMC-7721cells were 0.183, 0.212, or 0.321 and 0.269, 0.362, or 0.293, respectively (Figure 1C and 1D). The uniformly low combination indices (all < 0.5) further demonstrate the strong synergistic inhibition of cell viability. In order to test the long-term effect of DSF plus Aur co-treatment on cancer cells, we measured colony formation of SMMC-7721 and HepG2 cells in soft agar. As shown in Figure 1E, the co-treatment resulted in fewer colonies than the single-drug treatments after 7 days culture.

### DSF enhanced Aur-induced cell death

**Figure 2:**
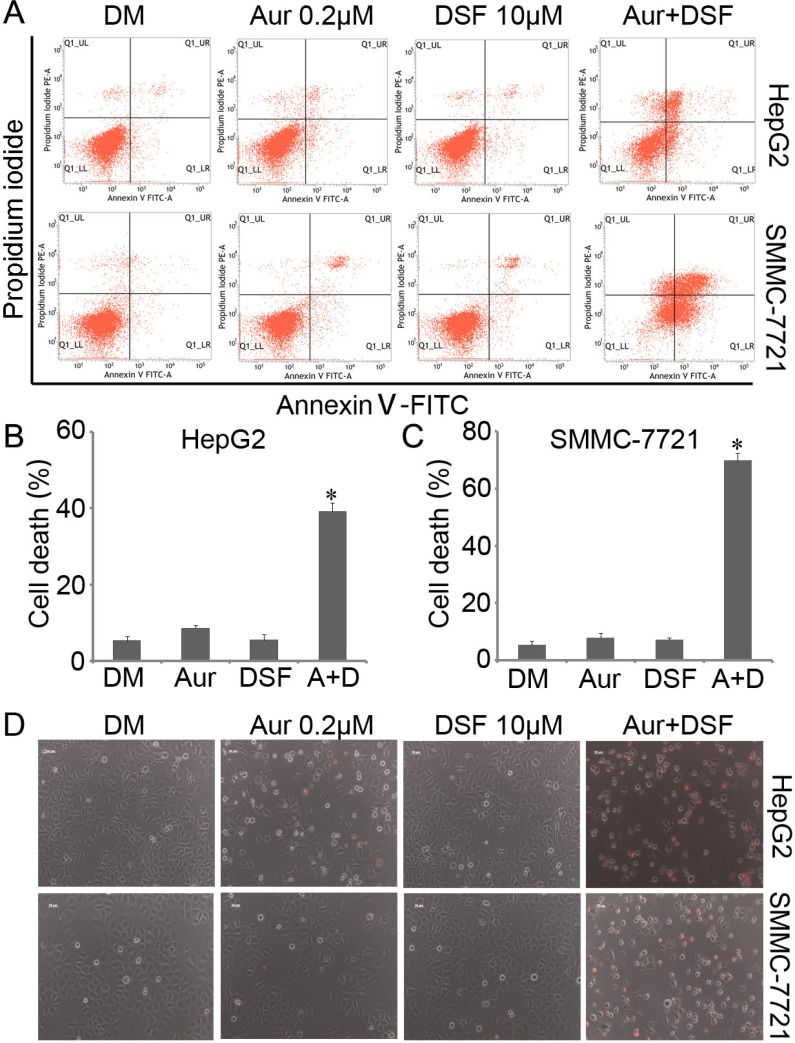
Aur and DSF synergistically induced cancer cell death (**A–C**) HepG2 or SMMC-7721 were seeded in 6-well plates and exposed to either Aur (0.2 μM), DSF (10 μM) or their combination for 24 h. The cultured cells were collected and stained with Annexin V FITC/propidium iodide (PI), followed by flow cytometry analysis. The representative images (A) and summary of cell death (B and C) are shown. Mean ± SD (*n* = 3). DM, DMSO. *P < 0.05 versus vehicle control. **(D)** HepG2 or SMMC-7721 were treated as (A) for 24 h, followed by direct PI staining in live cells, and then imaged by an inverted fluorescence microscope. The representative merged images are shown. Mean ± SD (*n* = 3).

To investigate whether DSF and/or Aur induced cell viability inhibition correlates with cell death, HepG2 and SMMC-7721 were exposed to either DSF (10 μM), Aur (0.2 μM) or their combination for 24 h. Cell death was detected using Annexin-V FITC and propidium iodide (PI) staining followed by flow cytometry and using PI staining followed by fluorescent microscopy in living cells. The flow cytometry study revealed that in both HepG2 and SMMC-7721 cells, less than 10% cell death was induced by either DSF or Aur respectively, while almost 40% (in HepG2) and 60%∼70% (in SMMC-7721) of cell death were induced by the co-treatment for 24 h (Figure 2A, 2B and 2C). The fluorescence microscopy showed that few PI-positive cells were induced by DSF or Aur alone but a significantly high percentage of PI-positive cells were induced by the DSF and Aur combined treatment (Figure 2D), indicating that the treatment with a combination of DSF and Aur significantly enhances cell death in hepatoma cancer cells.

### Induction of apptosis by DSF+Aur co-treatment is associated with caspase activation, decreased expression of anti-apoptotic proteins and increased expression of pro-apoptotic proteins

**Figure 3:**
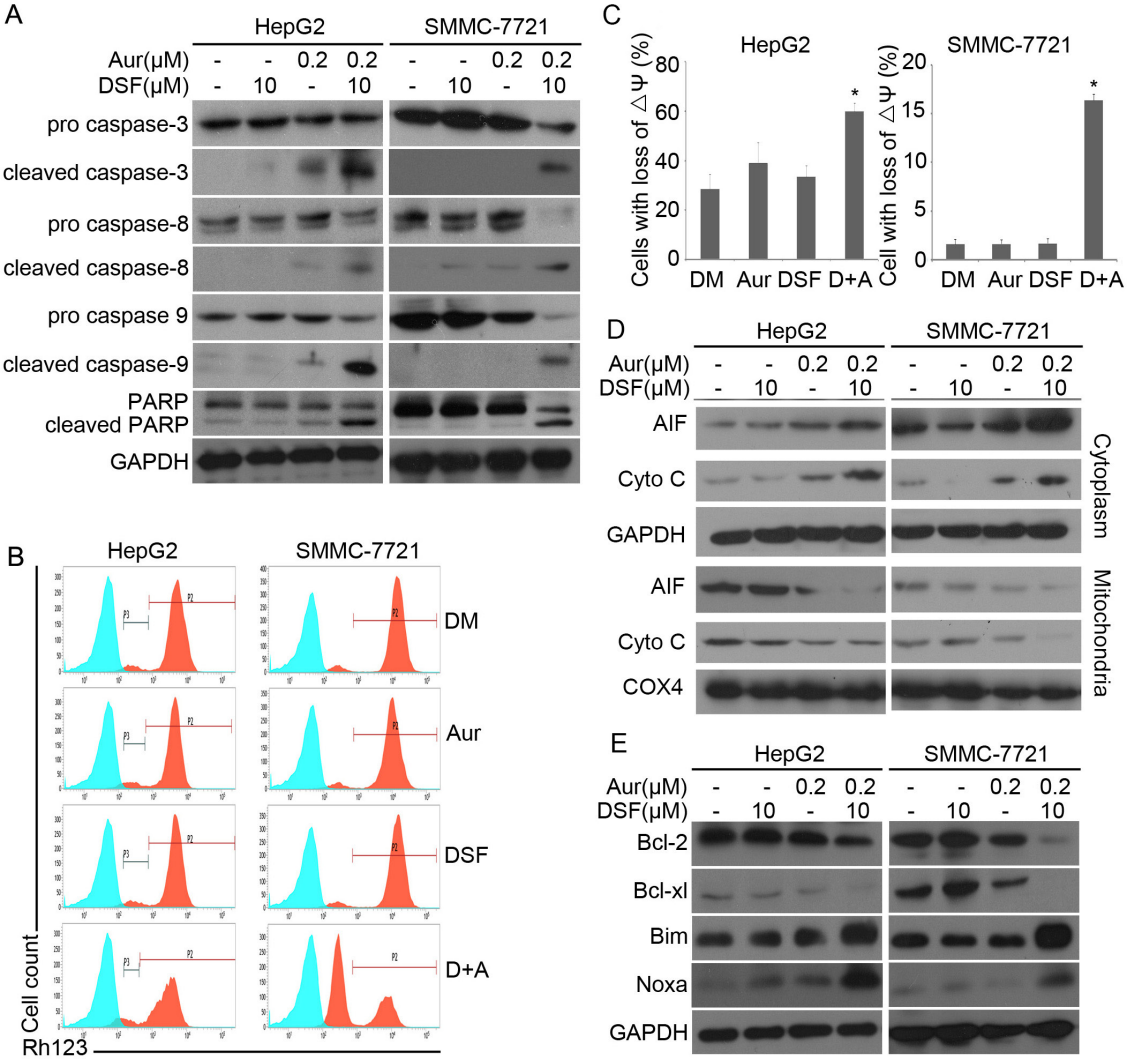
DSF and Aur co-treatment induced caspase activation and down-regulated expression of anti-apoptotic proteins **(A)** HepG2 or SMMC-7721 were treated with Aur (0.2 μM), DSF (10 μM), or their combination for 24 h. Total proteins were extracted from the cultured cells and subjected to western blot analysis using antibodies against pro- or cleaved caspase-3, -8 and -9, and PARP. GAPDH was used as a loading control. **(B)** and **(C)** HepG2 (left) or SMMC-7721 (right) were exposed to Aur (0.2 μM), DSF (10 μM), or their combination for 12 h. Mitochondrial membrane potential was detected by rhodamine-123 staining and flow cytometry. The proportion of cells with loss of ΔΨ was shown. Graphs represent data from three independent experiments. Mean ± SD (*n* = 3). *P < 0.05, compared with other treatments. **(D)** and **(E)** Cancer cells were treated as in (A), AIF and cytochrome C in the cytoplasm and mitochondria were analyzed with western blot (D). Total Bcl-2, Bcl-xl, Bim and Noxa were detected by western blot analysis (E).

We and others have reported that Aur, a clinically used anti-rheumatic agent, inhibits 19S DUBs and induces apoptosis associated with caspase activation and loss of MMP in various cancer cells [23]. Here, we investigated whether caspases and mitochondria associated signaling pathways were involved in the induction of apoptosis by the DSF and Aur combined treatment. It was found that the combination of DSF and Aur dramatically activated caspase-3,-8 and -9 and increased the cleavage of PARP (Figure 3A). It is widely accepted that mitochondria are the regulating center of apoptosis. As shown in Figure 3B and 3C, the integrity of mitochondrial membranes was decreased in both SMMC-7721 and HepG2 cells after co-treatment with DSF and Aur. The release of cytochrome C and apoptosis inducing factor (AIF) from mitochondria to the cytoplasm has been recognized as the early stage of apoptosis. To determine whether DSF+Aur co-treatment triggers the mitochondrial pathway, cancer cells were exposed to Aur, DSF and their combination for 12 hours. Cytosolic and mitochondrial fractions were extracted and the cytochrome C and AIF levels were detected by western blot analyses. As shown in Figure 3D, cytochrome C and AIF levels were highly elevated in the cytoplasm after DSF+Aur treatment, which indicates that DSF+Aur could activate the mitochondrial apoptosis pathway. Further supporting this observation, DSF and Aur synergistically decreased anti-apoptotic proteins Bcl-2 and Bcl-xl, and increased pro-apoptotic proteins Bim and Noxa.

### Aur and DSF synergistically induced unfolded protein response (UPR) and accumulation of ubiquitinated proteins (Ub-prs)

**Figure 4:**
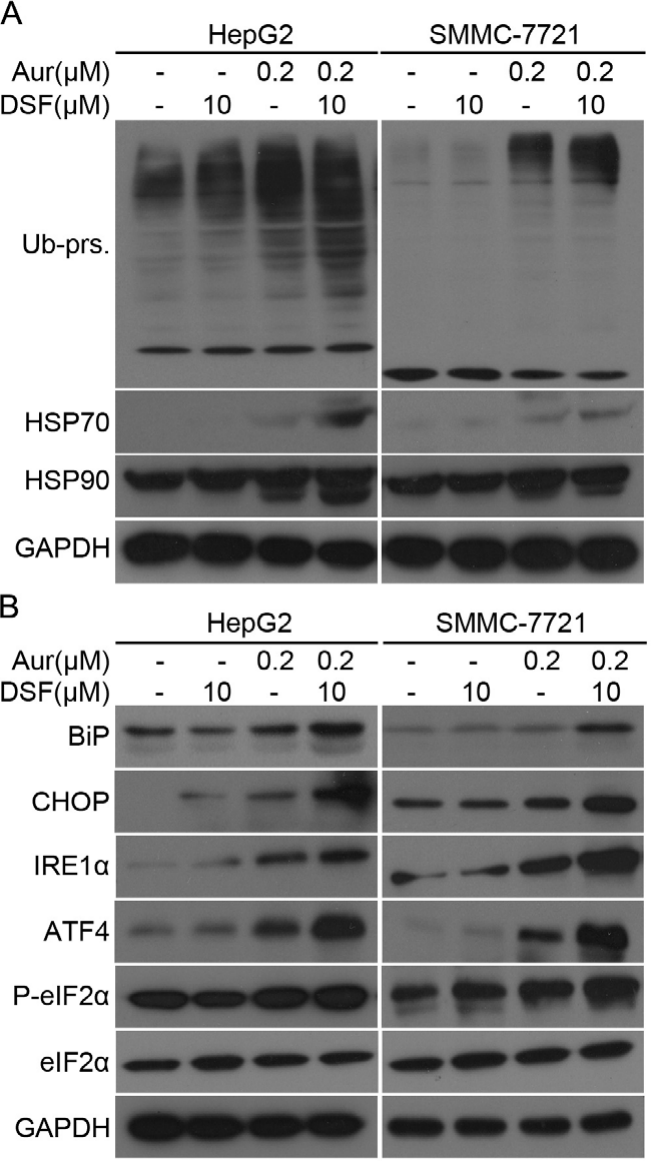
Aur and DSF combined treatment led to Ub-prs accumulation of ubiquitinated proteins and ER stress **(A)** HepG2 (left) or SMMC-7721 (right) were treated with Aur or/and DSF as indicated for 24 h. Ubiquitinated proteins (Ub-prs), HSP70, and HSP90 were detected by western blot analyses. GAPDH was used as a loading control. **(B)** Total proteins extracted from cancer cells treated as in (A), and ER stress related proteins Bip, CHOP, IRE1α, ATF4, phosphorylated eIF2α (P-eIF2α), and total eIF2α were detected by western blot analyses. Representative images were shown from three repeats.

Our previous reports have shown that Aur induces accumulation of Ub-prs due to inhibition of UCHL-5 and USP14 [23]. Hence, we tested whether DSF could enhance Aur-induced Ub-prs accumulation and UPR. We found that combination of the two agents significantly increased the protein expression of HSP70 and HSP90, accompanied by Ub-prs accumulation (Figure 4A). Moreover, we found that the combination treatment increased the expression of ER stress related proteins, including Bip, CHOP, IRE1α, ATF4 and P-eIF2α (Figure 4B). The above results indicate that the combination of DSF and Aur strongly enhanced Ub-prs accumulation and ER stress.

### The induction of caspase activation and PARP cleavage synergistically by Aur and DSF can be reversed by z-VAD-FMK and N-acetyl-cysteine (NAC)

**Figure 5:**
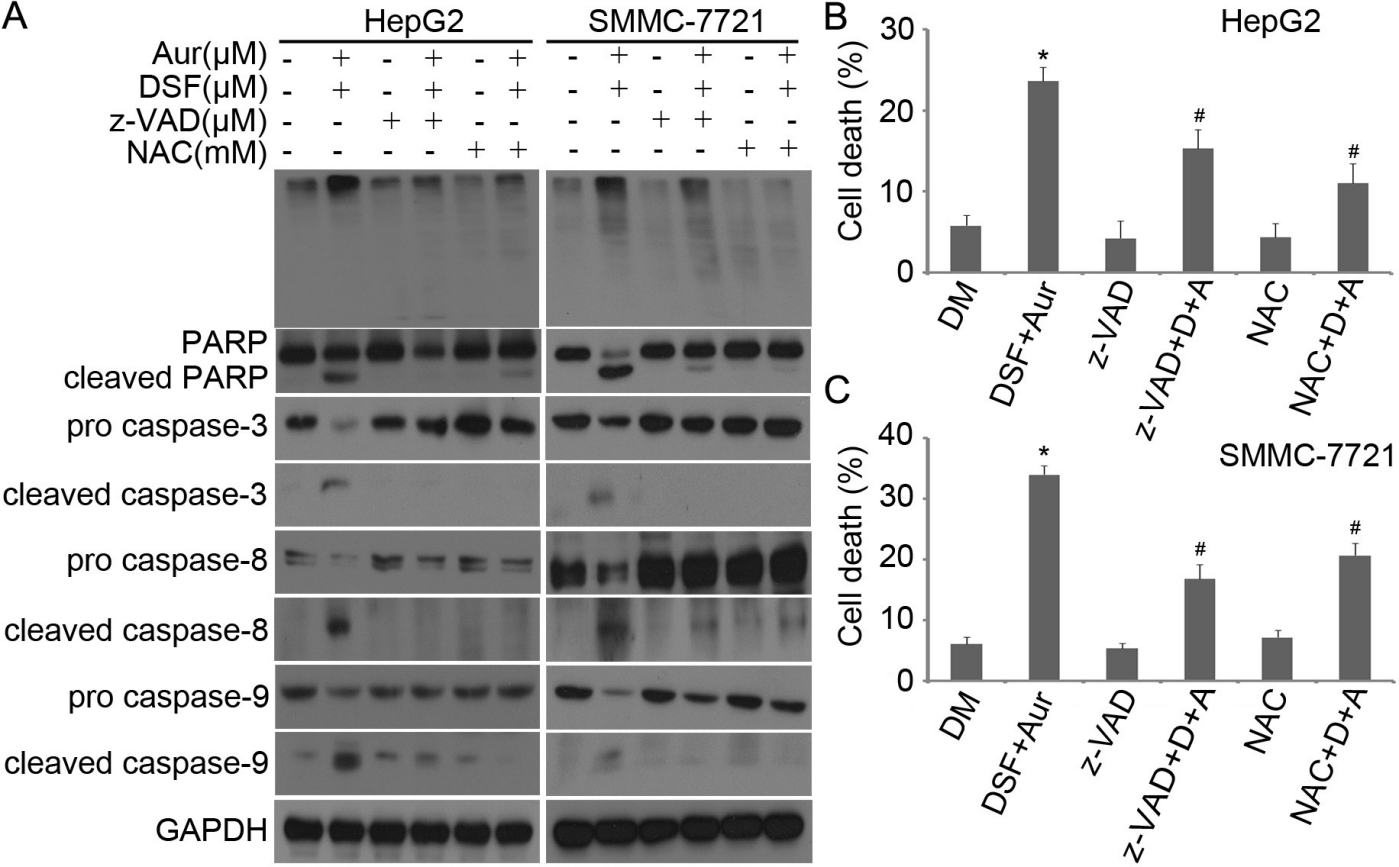
z-VAD-FMK and NAC prevented Aur + DSF from inducing capase activation and PARP cleavage **(A)** HepG2 (left) or SMMC-7721 (right) were exposed to the combination of Aur (0.2 μM) and DSF (10 μM) in the absence or presence of z-VAD-FMK (50 μM) or NAC (5 mM) for 18 h. Ubiquitinated proteins (top), PARP cleavage, pro- and cleaved caspase-3, -8 and -9 were detected by western blot analyses. Representative images of independent experiments are shown. GAPDH was used as a loading control. **(B)** and **(C)** HepG2 (upper) or SMMC-7721 (lower) cancer cells were treated with the combination of Aur and DSF in the absence or presence of z-VAD-FMK or NAC for 18 h. The treated cells were collected to stain with Annexin V FITC/PI, followed by flow cytometry. Data of three independent experiments are summarized and shown. Mean ± SD (*n* = 3). *P < 0.05 versus vehicle control; #*P* < 0.05 *versus* Aur + DSF.

In the experiments shown Figure 5, we observed in both HepG2 and SMMC-7721 cells that both pan-caspase inhibitor Z-VAD-FMK and the Aur active site blocker NAC almost completely blocked the activation of caspase-3,-8 and -9 and the cleavage of PARP; and NAC but not Z-VAD-FMK blocked Ub-prs accumulation (Figure 5A). Additionally, both Z-VAD-FMK and NAC prevented Aur+DSF co-treatment from inducing cell death (Figure 5B and 5C). These findings are consistent with the proteasome inhibition effects of Aur observed in our previous reports [23]. Taken together, these results demonstrate that Ub-prs accumulation, prior to caspase activation, is critical to the induction of cell death by the combined treatment.

### ROS generation is increased by Aur-DSF co-treatment but it is not required for the co-treatment to induce cell death

**Figure 6:**
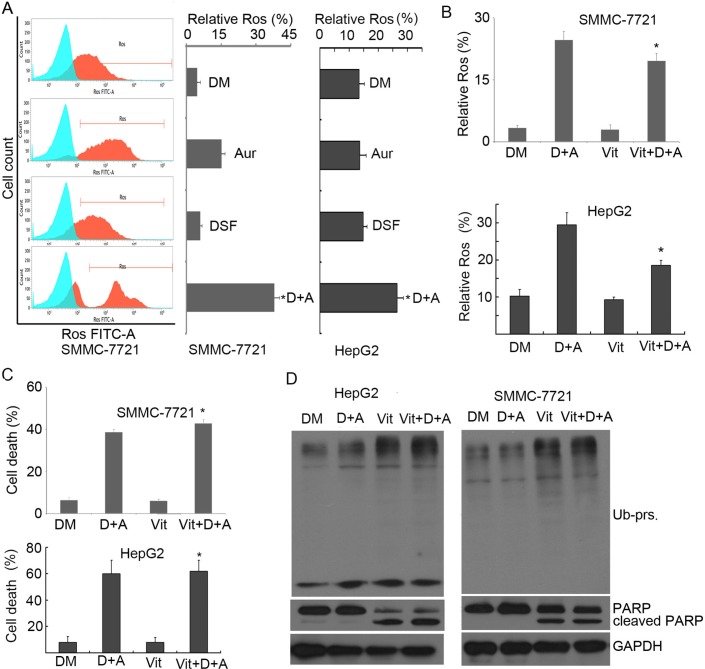
Combination of Aur and DSF resulted in ROS generation but ROS was not responsible for apoptosis **(A)** SMMC-7721 and HepG2 cells were treated with Aur (0.2 μM), DSF (10 μM), or their combination for 12 h. ROS generation was detected by flow cytometry. Relative ROS was shown. Mean ± SD (*n* = 3). *P < 0.05, compared with other treatments. **(B)** SMMC-7721 and HepG2 cells were treated with the combination of Aur (0.2 μM) and DSF (10 μM) in the absence or presence of Vitamin C (100 μM) for 12 h. ROS generation was detected and shown. *P < 0.05, compared with D + A treatment. **(C)** SMMC-7721 and HepG2 cells were treated as in (B). Cell death was assessed by flow cytometry from three independent repeats. The summarized data are shown. *P < 0.05, compared with D + A treatment. **(D)** SMMC-7721 and HepG2 cells were exposed to co-treatment of Aur (0.2 μM) and DSF (10 μM) as in (B). Ubiquitinated proteins (Ub-prs) and PARP cleavage were detected by western blot. GAPDH was used as a loading control.

It had been reported that Aur could induce ROS generation in various cancer cells by inhibiting TrxR and the ROS generation was thought to be responsible for cell death Induction by Aur. However, we previously observed that Tbhq could completely scavenge Aur-mediated ROS production but could not block Ub-prs accumulation and cytotoxicity [23]. Likewise, here we found that DSF and Aur synergistically enhanced ROS production (Figure 6A), which was blocked by using another antioxidant agent, Vitamin C (100 μM; Figure 6B); however, similarly to our prior report, the scavenging of ROS by Vitamin C failed to block cell death (Figure 6C), Ub-prs accumulation, or PARP cleavage (Figure 6D) induced by the DSF and Aur co-treatment. These findings further confirm that DSF and Aur combination induces apoptosis through DUB inhibition, not by ROS generation.

### Aur and DSF combination exhibits anti-cancer activity *in vivo*

**Figure 7:**
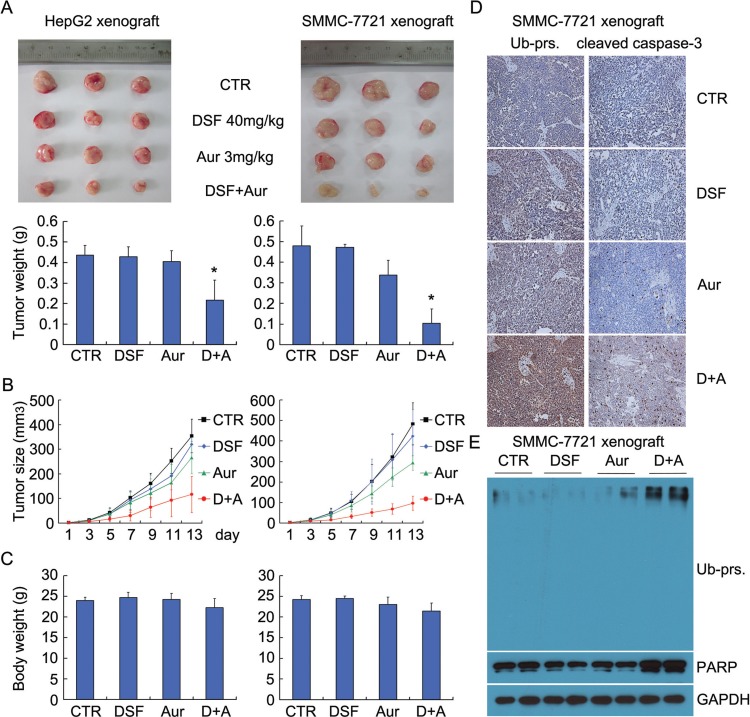
Aur and DSF co-treatment inhibited tumor growth *in vivo* **(A)** BALB/c nude mice bearing HepG2 or SMMC-7721 xenografts were treated with DSF (40 mg/kg/d,i.p.), Aur (3 mg/kg/day, i.p.), or their combination for 15 days. Xenograft images and xenograft weight are shown. Mean ± SD. *P < 0.05 versus each treatment. **(B)** and **(C)** Tumor size (B) and body weight (C) were recorded every two days. Summarized data are shown. **(D)** Representative micrographs of immunohistochemistry staining for total ubiquitinated proteins (Ub-prs) and cleaved caspase-3 in nude mouse tumor tissues. All the immunostaining was repeated in three mouse tumor tissues and the images shown were collected at a magnification of 200 ×. **(E)** Ubiquitinated proteins and PARP cleavage were detected by western blot analyses with three independent experiments. Representative images are shown. GAPDH was used as a loading control.

Given that our *in-vitro* experiments show a promising synergisticanti-cancer activity by DSF and Aur on human hepatoma cell lines HepG2 and SMMC-7721, we next evaluated the *in vivo* effect of DSF and Aur combination using nude mouse xenograft models. We found that tumor weight and tumor size of nude mouse models in the combinational treatment group were significantly reduced, compared with each single-agent treatment group (Figure 7A and 7B), while there were no significant differences in body weight among four groups (Figure 7C). The immunostaining results showed that the representative proteasome substrates, Ub-prs, and activated caspase-3 proteins were all significantly increased (Figure 7D) in the DSF and Aur combination -treated tumors. Similarly to the immunostaining results, western blot results showed that treatment with the DSF and Aur combination significantly increased the levels of Ub-prs and cleaved PARP (Figure 7D). Together, these results demonstrate that DSF and Aur combination selectively inhibits proteasome function and tumor growth *in vivo*.

## DISCUSSION

DUBs are emerging as a novel target for anticancer strategy. Recently, a growing number of DUB inhibitors were discovered and designed to be anti-cancer agents. As a clinically used anti-rheumatic arthritis drug, Aur, possesses excellent anticancer property and has proven to be a promising anticancer agent [20–22]. However, the underlying mechanisms remain less clear. We have recently demonstrated that Aur mainly targets proteasome-associated UCHL5 and USP14 and thereby induces proteasome inhibition, caspase activation and apoptosis in several cancer cell lines [23, 24]. To enhance selectivity and lower the potential toxic side effects of Aur on normal tissue, we further sought to search for agents that can synergistically increase the anticancer effect of Aur. Recent reports have indicated that DSF has antitumor and chemosensitizing activities. Our present study reveal that (1) DSF synergistically enhances the cytotoxicity of Aur, leading to cell death in human hepatoma HepG2 and SMMC-7721 cancer cells; (2) cell death induced by DSF+Aur co-treatment is dependent on inhibition of the 19S proteasome-associated, caspase activation and ER stress; (3) blocking caspase activation by z-VAD-FMK and blocking proteasome inhibition by NAC prevented the induction of cell death by DSF + Aur co-tretament.

It is known that DSF not only acts as an aldehyde dehydrogenase inhibitor for alcoholism treatment but also potentiates the effect of many chemotherapeutic agents. In the current study, DSF (within 40 μM or 40 mg/kg/d) itself does not induce cytotoxicity, proteasome inhibition, or apoptosis in cultured human hepatoma cancer cells or xenograft models. Aur at rather low doses could only induce modest cytotoxicity and proteasome inhibition but these effects were dramatically exacerbated by co-treatment with DSF. These findings suggested that DSF or its metabolites can synergistically enhance Aur induction of cytotoxicity and proteasome inhibition directly or indirectly.

Activation of the caspases cascade is the key event of apoptosis. Activated caspase-3 cleaves death substrate PARP to generate a specific 85 kDa apoptotic fragment, which leads to apoptosis [32]. DSF and Aur combination induced activation of caspase-8, caspase-9, caspase-3 and PARP cleavage, suggesting that both the extrinsic and the intrinsic pathways were involved in the apoptosis. We further investigated the underlying molecular mechanism. DSF and Aur combined treatment induced the down-regulation of anti-apoptotic proteins like Bcl-2 and Bcl-xL and the enrichment of pro-apoptotic proteins such as Bim and Noxa, followed by the loss of mitochondria membrane potential (ΔΨ) and thus activation of caspase-9. In addition, we further confirmed that the combination of DSF and Aur induced proteasome inhibition and increased the expression of HSP70 and HSP90 and a series of ER stress associated proteins such as Bip, CHOP, IRE1α, ATF4, and P-eIF2α. The unfolded protein response (UPR) is the cellular response to ER stress and contributes to cancer survival or death [33]. Generally, GRP78/Bip binds to PERK and leads to the phosphorylation of eIF2α on serine residue 51, which inhibits the translation of mRNA [34], and paradoxically increases translation of ATF4 [35]. On one hand, ATF4 regulates transcription of pro-survival genes; on the other hand, ATF4 upregulates the expression of pro-death transcription factor C/EBP homology protein (CHOP) [33, 36]. Based on our data, we hypothesize that there might be another pathway involved in the induction of apoptosis by the combined treatment: the DSF and Aur combination first cause proteasome inhibition, which then leads to accumulation of damaged and unfolded proteins in the ER, resulting in ER stress and thereby sustained UPR and caspase activation.

We have previously confirmed that NAC, a classical ROS inhibitor, can bind to the active site of Aur and block its inhibiting effects on the proteasomal DUBs. In the current investigation, NAC completely prevented DSF and Aur co-treatment from inducing proteasome inhibition, caspase activation and cell death. To further distinguish whether ROS generation, DUBs inhibition or both are required for the cell death induction by the combined treatment, here we employed another antioxidant agent Vitamin C, which would not change the effect of Aur but can rapidly and efficiently eliminate cellular ROS. We found that Vitamin C successfully scavenged ROS generation but failed to block Ub-prs accumulation, PARP cleavage and cell death. This further confirms that the induction of apoptosis by DSF and Aur combined treatment depends on DUB inhibition rather than ROS generation.

By treating nude mice with human hepatoma HepG2 and SMMC-7721 xenografts, we have also demonstrated here that DSF and Aur show synergistic anticancer effects *in vivo*. DSF and Aur co-treatment significantly inhibited tumor growth, accompanied by Ub-prs accumulation, PARP cleavage, an activation of caspase 3. Notably, we have also found that the co-treatment did not inhibit the body weight while exhibited a remarkable antitumor and pro-apoptotic activity in the xenograft model.

Originally developed as an anti-rheumatoid arthritis drug, Aur is actually an inhibitor of proteasomal DUBs whereas DSF is an inhibitor of aldehyde dehydrogenase for treating alcoholism; both agents have been used clinically for several decades and only recently reported to possess anti-tumor properties. In the current study, we have unraveled a model of synergism between Aur and DSF in the induction of apoptosis in hepatoma cancer cells and xenografts, providing a potentially novel anticancer strategy that should be relatively easy to be translated to the clinic as both are in clinical use for treatment of other disorders.

## MATERIALS AND METHODS

### Materials

Aur and pan caspase inhibitor z-VAD-FMK were obtained from Enzo Life Sciences International, Inc. (Plymouth Meeting, PA) and dissolved in DMSO at a stock concentration of 10 mM, aliquoted and stored at −80°C. DSF was acquired from Sigma-Aldrich (St. Louis, MO). MTS assay (CellTiter 96 Aqueous One Solution reagent) was purchased from Promega Corporation (Madison, WI, USA). PI and Annexin V-FITC apoptosis Detection Kit, DCFH-DA and cell apoptosis Rhodamine 123 Detection Kit were purchased from Keygen Company (Nanjing, China). Antibodies (Abs) used in this study were purchased from following sources: anti-ubiquitin (P4D1) (Santa Cruz Biotechnology, Santa Cruz, CA); anti-PARP, anti-BIP (C50B12), eIF2α, Phospho-eIF2α(Ser51), anti-IRE1α (14C10), anti-caspase-3 (8G10), anti-Bcl-2 (50E3), anti-Bcl-xl (54H6), anti-CHOP (L63F7), anti-HSP70, anti-HSP90, anti-caspase-8 (1C12), and anti-caspase-9 (C9) (Cell Signaling Technology, Beverly, MA, USA); anti-cleaved caspase-8 (Cleaved Asp384) (Assay biotechnology Company, Inc); anti-cleaved caspase-9 p35 (D315), anti-cleaved caspase-3 (p17), anti-Bim (Y36), anti-ATF4 (R239), anti-Noxa (EPR9735 [B]), and anti-GAPDH (Bioworld Technology, Inc).

### Cell lines and cell culture

Human hepatoma cell lines HepG2 and SMMC-7721 were purchased from American Type Culture Collection (Manassas, VA, USA) and grown in RPMI 1640 supplemented with 10% FBS. Cultured cells were maintained at 37°C and 5% CO_2_.

### Cell viability assay

MTS assay (CellTiter 96 Aqueous One Solution reagent) was used to test cell viability as we previously reported [37]. In brief, exponentially growing HepG2 or SMMC-7721 cells were seeded at 2500 cells/well in a 96-well plate. After incubation for 24 h, cells were treated with Aur and/or DSF, followed by continuous incubation for 48 h. 20 μl MTS was directly added to each well and the incubation was continued for an additional 3 h. The absorbance of optical density was measured with a microplate reader (Sunrise, Tecan) at wavelength 490 nm. Cell viability was calculated by the following formula: cell viability (%) = (average absorbance of treated group - average absorbance of blank)/(average absorbance of untreated group - average absorbance of blank) ×100%.

### Cell death assay

Apoptosis assay was performed according to previous description [38]. Briefly, cultured HepG2 and SMMC-7721 cells were harvested and washed with 4°C PBS twice and resuspended with the binding buffer, followed by Annexin V-FITC incubation for 15 min and PI staining for another 15 min in dark. The stained cells were analyzed with flow cytometry within 30 min. To monitor temporal changes in the incidence of cell death in the live culture condition, HepG2 and SMMC-7721 cells were seeded into 6-well plates and PI was added directly to the cell culture medium, then the cells in the dish were kinetically imaged with an inverted fluorescence microscope equipped with a digital camera (Axio Obsever Z1, Zeiss).

### Western blot analysis

Western blot analysis was performed as we described previously [39]. In brief, equal amounts of total proteins extracted from cultured cells were separated by 12% SDS–PAGE and transferred to polyvinylidene difluoride (PVDF) membranes. The blots were blocked with 5% milk for 1 h. Primary Abs and horseradish peroxidase (HRP)-conjugated secondary Abs were each incubated for 1 h. The bounded secondary antibodies were reacted to the ECL detection reagents and exposed to X-ray films (Kodak, Japan).

### Measurement of ROS generation

Cancer cells were treated with Aur and/or DSF for 12 h, and then the cells were incubated with the serum-free medium with addition of 10 μM of DCFH-DA for 20 min at 37°C. Following the staining, the cells were washed with 4°C PBS twice, and then collected for flow cytometry analysis. The fold changes of mean fluorescence intensities were shown in the diagram. Mean values and standard deviations were calculated from triplicates.

### Clonogenic assay

This assay was performed as we previously described [40]. HepG2 and SMMC-7721 cells exposed to Aur (0.2 μM), DSF (10 μM) or their combination for 12 h were suspended in 30% agarose supplemented with 20% FCS and 50% RPMI-1640 medium then cultured in 60 mm dishes in an atmosphere of 5% CO_2_ for 7 days, then stained with 0.3% crystal violet solution. The colonies > 60 μm were counted under a light microscope. The experiments were done in triplicate.

### Mitochondrial membrane integrity measurement

The mitochondrial membrane potential of Aur and/or DSF -treated and untreated cells was assayed by using Rhodamine-123 staining as we previously reported [40]. Cells were treated with Aur and/or DSF for 12 h and stained with 1 μM of Rhodamine-123 for 30 min at 37°C. Following the staining, the cells were washed with 4°C PBS twice, and then harvested for flow cytometry analysis. Mean values and standard deviations were calculated from triplicates.

### Nude mouse xenograft model

Male Balb/c nude mice aged 5 weeks were purchased from Guangdong Animal Center and housed in the animal facility of Guangzhou Medical University approved by the Guangdong Animal Center. The mice were housed in barrier facilities with a 12 h light dark cycle, with food and water available ad libitum. Balb/c mice were inoculated subcutaneously in the left armpit of each mouse with HepG2 or SMMC-7721 cells (1 × 10^6^ cells/mouse) respectively. After 72 h of inoculation, mice were randomly divided into 4 × 2 groups and i.p. injected with either vehicle (10% DMSO, 30% Cremophor EL and 60% normal saline) or Aur (3 mg/kg/day) and/or DSF (40 mg/kg/day) for totally 15 days respectively. Tumors were measured every other day with use of calipers. Tumor volumes were calculated as previously reported. Aur and DSF were dissolved in the buffer with 10% DMSO, 30% Cremophor EL and 60% normal saline.

### Immunohistochemical staining

Formalin-fixed xenografts were embedded in paraffin and sectioned according to standard techniques as we previously reported [40]. Tumor xenograft sections (4 μm) were immunostained using the MaxVision kit (Maixin Biol) according to the manufacturer's instructions. The primary antibodies were against ubiquitin and cleaved caspase 3. 50 μl MaxVisionTM reagent was applied to each slide. Color was developed with 0.05% diaminobenzidine and 0.03% H2O2 in 50 mM Tris-HCl (pH 7.6), and the slides were counterstained with hematoxylin. A negative control for every antibody was also included for each xenograft specimen by substituting the primary antibody with preimmune rabbit serum.

### Combination index

The interaction between the two compounds was quantified by determining the combination index (CI). The CI was calculated using the Chou-Talalay equation [41]. The general equation for the classic isobologram is as follows: CI *=* (D) 1/(Dx) 1 + (D) 2/(Dx) 2. Dx indicates the dose of one compound alone required to produce an effect, and (D) 1 and (D) 2 are the doses of compounds 1 and 2, respectively, necessary to produce the same effect in combination. CI < 1 indicates synergism; CI *=*1 indicates an additive effect; and CI > 1 indicates antagonism.

### Statistical methods

Mean ± SD are presented where applicable. Unpaired Student's *t*-test or one way ANOVA is used where appropriate for determining statistic probabilities. GraphPad Prism4.0 software (GraphPad Software) was used for statistical analysis. *P* value less than 0.05 was considered statistically significant.
